# 

**Published:** 1885-09

**Authors:** 


### CONTENTS.

ORIGINAL ARTICLES: *Pages.*

A Case of Lupus Exedens of Face. By T. C. Osborn, M. D., Cleburne, Texas .... ........ 109

Gonnorrhœal Ophthalmia. By T. D. Wooten, M. D., Austin. ...................................... 117

Etiology of Tubercular Disease. By J. W. McLaughlin, M. D., Austin ... ................ 126

New Operation and Instrument for Fistula in Ano. By J. A. Gibson, M. D., Foster, Texas....... 136

A Long Cord. By R. G. Williams, M. D., Whitney, Texas.................................. 137

EDITORIAL:

Principle vs. Policy: Consistency vs. Expediency 138

Finish the Good Work—A Plan Suggested to regulate the Practice .............................. 144

Quarantine on the Rio Grande.................... 146

The Destruction of Shenanegan.................. 147

CULLINGS FROM CONTEMPORARIES:

Successful Laparotomy for Gunshot Wound of Intestines ..................... ............. 148

Episiotomy—Traumatic Tetanus—Worth Knowing 149–150

SOCIETY NOTES:

Transactions T. S. M. A.—Travis County Medical Society—Physicians' Mutual Benefit Association of Texas. ............................ 150–151

MELANGE:

Being a Miscellany of Editorial Notes, News, and other Items of Interest..................... 151–155

ADVERTISERS' EDITORIALS ......................... 156–157

